# Osmotic Demyelination Syndrome Complicating Moderate Hyponatremia Correction in the Emergency Department: A Case Report

**DOI:** 10.1002/ccr3.71222

**Published:** 2025-10-16

**Authors:** Khaled Alsa'ed, Soud Deek, Gi Eun Kim, Muhammad Faisal Wadiwala, Anwar I. Joudeh

**Affiliations:** ^1^ Department of Internal Medicine Hamad General Hospital, Hamad Medical Corporation Doha Qatar; ^2^ Department of Neurology Al Khor Hospital, Hamad Medical Corporation Doha Qatar; ^3^ Department of Internal Medicine Al Khor Hospital, Hamad Medical Corporation Doha Qatar

**Keywords:** central pontine myelinosis, hyponatremia, magnetic resonance imaging, osmotic demyelination syndrome

## Abstract

Osmotic demyelination syndrome (ODS) is a rare neurological complication that can be associated with rapid correction of hyponatremia. This report describes a middle‐aged male patient with a chronic alcoholism history who developed ODS following moderate hyponatremia correction with 1.5 L of isotonic saline in the emergency department. Initially, the patient presented with tremors and ataxia, which later progressed to lower limb weakness and slurred speech. An initial MRI of the brain showed pontine diffusion restriction, which was misinterpreted as acute ischemic stroke. However, follow‐up imaging confirmed central pontine lesions, consistent with ODS. Despite physiotherapy, the patient developed complications, including subdural hematoma, and succumbed to cardiac arrest. This case highlights the risk of ODS even with moderate hyponatremia and underscores the need for cautious sodium correction, close monitoring, and thorough evaluation to prevent misdiagnosis and associated adverse outcomes. It emphasizes the importance of vigilance in managing high‐risk patients to avoid this potentially fatal condition.

**Summary**

- Correction of even moderate hyponatremia in high‐risk patients (e.g., chronic alcoholism, hypokalemia, and malnutrition) requires cautious fluid administration and close monitoring of sodium levels during and after correction.
- A high index of suspicion should be held in the following weeks for ODS, with timely neuroimaging for diagnosis.

## 1 Introduction

Osmotic demyelination syndrome (ODS), formerly known as central pontine myelinolysis, is a severe iatrogenic neurological condition associated with significant morbidity and mortality. It is most observed following overly rapid correction of chronic hyponatremia, particularly when serum sodium levels are ≤ 120 mmol/L [1]. Sodium is the principal electrolyte regulating plasma osmolarity and, consequently, serum tonicity. Hyponatremia is defined as a serum sodium concentration below 135 mmol/L and is categorized as moderate (125–129 mmol/L) or severe (< 125 mmol/L) [2].

**FIGURE 1:**
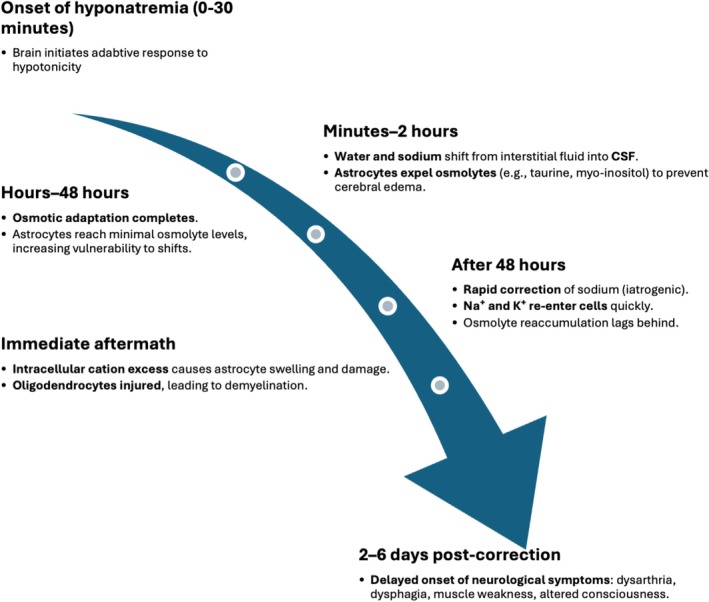
Timeline of pathophysiological events in osmotic demyelination syndrome (ODS). This timeline illustrates key events in ODS development following rapid correction of chronic hyponatremia. Initial brain adaptation involves water and sodium (Na^+^) shifting from the interstitial space to cerebrospinal fluid (CSF), and astrocytes expelling osmolytes to prevent cerebral edema. These adaptations complete within 48 h. Rapid correction causes sodium and potassium (K^+^) to shift into cells faster than osmolyte reaccumulation, leading to intracellular osmotic stress, astrocyte and oligodendrocyte injury, and demyelination. Clinical symptoms typically emerge 2–6 days later. Abbreviations: ODS, Osmotic Demyelination Syndrome; Na^+^, sodium; K^+^, potassium; CSF, cerebrospinal fluid.

In hypotonic hyponatremia, reduced serum tonicity draws water into brain cells, increasing the risk of cerebral edema and neurological symptoms, especially when sodium levels decline rapidly. The brain adapts within minutes, moving sodium and water from the interstitial space to the cerebrospinal fluid and actively extruding osmolytes by astrocytes to reduce intracellular osmolality. These adaptations usually complete within 48 h [3]. However, when serum sodium is rapidly corrected, sodium and potassium exceed osmolyte reaccumulation, causing an intracellular osmotic imbalance. This influx of cations induces cellular stress, injuring astrocytes and oligodendrocytes and leading to demyelination in vulnerable brain regions [4], as illustrated in Figure 1.

ODS is challenging to diagnose due to its delayed onset, typically 2–6 days after rapid sodium correction, and its nonspecific symptoms, including dysarthria, dysphagia, muscle weakness, and impaired consciousness [5]. It's commonly associated with overly rapid correction of severe chronic hyponatremia, especially with hypertonic saline [6]. However, this case involved moderate hyponatremia (Na^+^ 125 mmol/L) corrected over several days with isotonic saline (1.5 L), reaching 134 mmol/L by day 5. While rare, ODS after moderate hyponatremia correction has been reported, especially in patients with risk factors like chronic alcoholism, hypokalemia, and malnutrition [7, 8, 9]. A 2023 population‐level study found fewer than 5% of ODS cases occurred with initial sodium ≥ 125 mmol/L, emphasizing its rarity [10]. This case highlights the risk of ODS despite isotonic saline and moderate correction rates, underscoring individualized risk stratification.

## 2 Case History/Examination

A 47‐years‐old man with chronic alcoholic use presented 2 weeks before with abdominal pain and vomiting 4–5 times for 3 days. Laboratory investigations showed lipase of 603 U/L (reference range: 13–60), sodium of 125 mmol/L (133–146), potassium of 3.2 mmol/L (3.5–5.3). His last sodium reading 9 months ago was 140 mmol/L. US abdomen showed gallbladder sludge with no acute cholecystitis. He was given 1.5 L of isotonic saline (lactated ringer's and sodium chloride 0.9%) and discharged the same day with follow‐up appointment in the clinic. Repeated laboratory tests done 5 days later showed sodium of 134 mmol/L and potassium of 3.4 mmol/L, and he was doing well clinically.

In 2 weeks, he came to the emergency department with sudden onset of heavy chest pain for 4 days, not associated with shortness of breath or palpitations. He also had abdominal pain recurrence for 4 days with vomiting once. On physical examination, he was alert and oriented, and his vital signs were within the normal range. He had postural and intentional tremors in his arms, truncal ataxia, and his power in lower limbs was 4‐/5.

## 3 Differential Diagnosis, Investigations, and Treatment

Laboratory tests were remarkable for high lipase level, sodium of 132 mmol/L, and low phosphorus (Table 1). Computed tomography pulmonary angiography showed segmental pulmonary embolism. He was initially treated as a case of alcoholic withdrawal syndrome with acute pancreatitis and pulmonary embolism, with lorazepam, intravenous fluids, and rivaroxaban.

**TABLE 1:** Summary of laboratory investigations at presentation, day 5 and day 17.

	Baseline	Day 5	Day 17	Reference range
White blood cells	4.6	—	4.1	4–10 × 10^−3^/μL
Sodium	125	134	132	133–146 mmol/L
Potassium	3.2	3.4	3.7	3.5–5.3 mmol/L
Phosphorus	—	—	0.35	0.8–1.5 mmol/L
Total bilirubin	29	—	27	0–21 μmol/L
Alanine transaminase	46	—	56	0–41 U/L
Aspartate transaminase	68	—	83	0–40 U/L
Alkaline phosphatase	74	—	106	40–129 U/L
Lipase	603	—	247	13–60 U/L
C‐reactive protein	9.4	—	4.8	0–5 mg/L

Two days into admission, the patient was still unable to walk, and he had persistent peripheral and truncal ataxia with slurred speech. Magnetic resonance imaging (MRI) of the brain showed a small T2 and FLAIR hyperintense lesion involving the central part of the pons with restriction in diffusion weighted images. These findings were first reported as acute infarction although it was mentioned that the images had a lot of artifacts. Given the recent history of pulmonary embolism, other investigations were done to rule out embolic stroke including transthoracic and transesophageal echocardiograms, as well as Holter monitor which were all reported as normal.

Due to the poor quality of the initial MRI images and a high clinical suspicion for osmotic demyelination syndrome (ODS), a repeat MRI of the head with contrast was performed on day 8 of admission. This imaging revealed an ovoid lesion in the central pons, which appeared hypointense on T1‐weighted sequences and hyperintense on T2‐weighted and FLAIR images, with no evidence of mass effect. The lesion demonstrated mild to moderate hyperintensity on diffusion‐weighted imaging with corresponding low apparent diffusion coefficient (ADC) values. It spared the ventrolateral pons and exhibited no gadolinium enhancement, presenting with the characteristic “batwing” appearance.

## 4 Conclusion and Results

**FIGURE 2:**
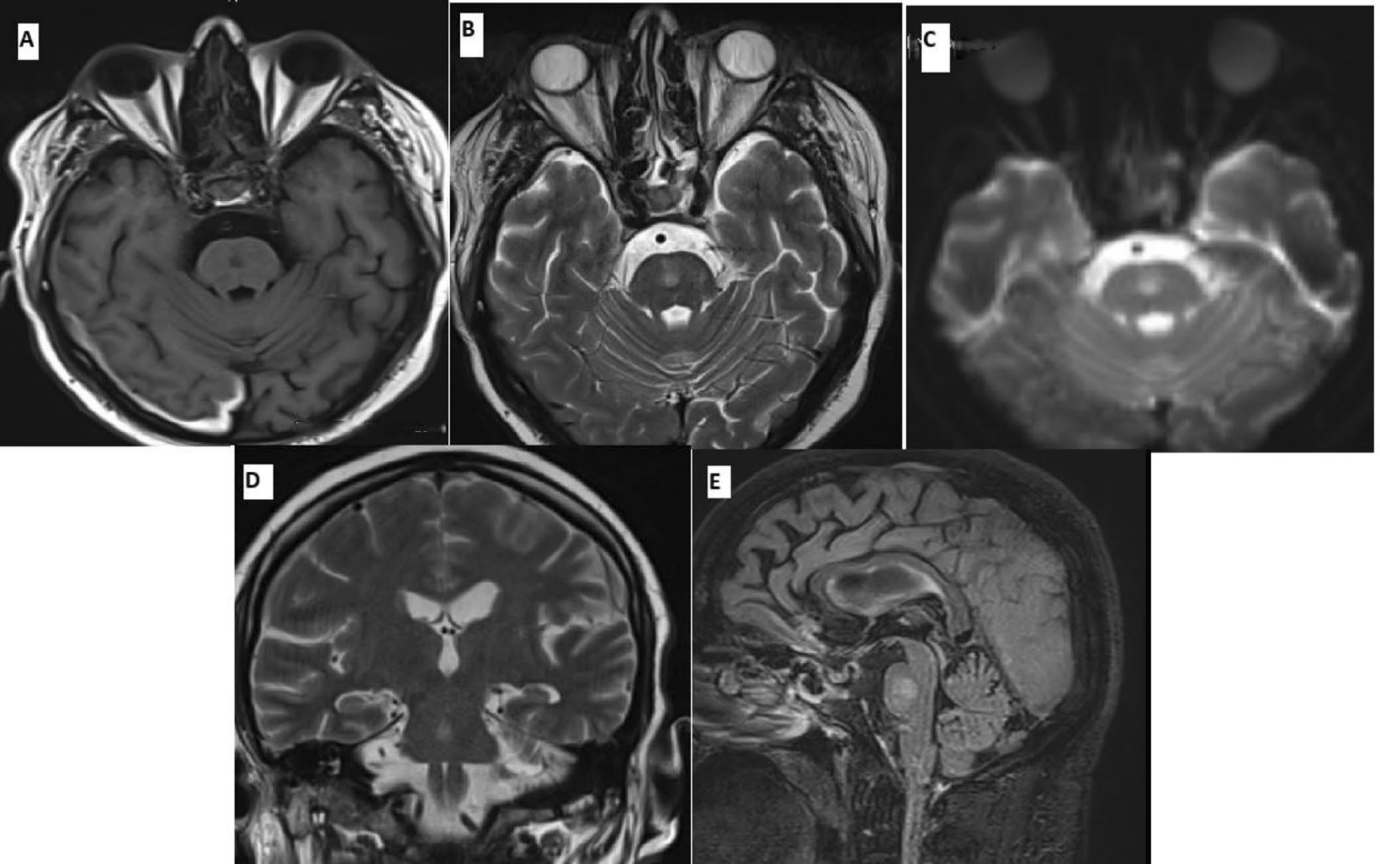
Magnetic resonance imaging (MRI) of the brain demonstrating an ovoid, slightly asymmetric lesion in the central pons. The lesion appeared hypointense on T1‐weighted images (A), hyperintense on T2‐weighted (B, D) and FLAIR images (E), and showed moderate brightness on diffusion‐weighted imaging (DWI) (C). Post‐contrast imaging revealed no gadolinium enhancement. The lesion spared the ventrolateral pons and possibly the corticospinal tracts. These findings are consistent with osmotic demyelination syndrome involving the pons, rather than an acute infarct. Additionally, there is a focal subdural hematoma measuring 8.7 mm in thickness along the right parietal convexity, which appears hyperintense on T1‐weighted images (A).

These findings were consistent with osmotic demyelination syndrome involving the pons, rather than an acute infarction. The repeated MRI also showed incidental right parietal subdural hematoma of 8.7 mm thickness without significant mass effect (Figure 2).

The patient underwent active physiotherapy during hospitalization, and he was improving to being able to walk with a walker. However, 10 days into admission, he developed disorientation and hallucinations, for which CT head was repeated, showing an increase in the subdural hematoma to 18 mm with midline shift of 22 mm to the left. The neurosurgery team took him for emergency right decompressive craniectomy, and he had a prolonged stay in the neurology intensive care unit afterwards. Unfortunately, he was pronounced brain dead and died from cardiac arrest approximately 1 month into admission.

## 5 Discussion

We presented an interesting case of ODS developing after moderate hyponatremia correction with a controlled amount of isotonic fluid infusion. The diagnosis of ODS was initially overlooked due to chronic alcoholism and was later mistakenly diagnosed as a pontine stroke. Typical findings on the repeated MRI of the brain in the setting of recent hyponatremia correction confirmed the diagnosis of ODS. ODS is an ominous disease with a protean range of clinical and radiological manifestations. The development of this syndrome in patients with only mild hyponatremia emphasizes the need for a thorough understanding of the risk factors, pathophysiology, and management strategies to prevent this potentially life‐threatening complication.

The initial sodium level in this case was 125 mmol/L. While most reported cases of osmotic demyelination syndrome (ODS) involved initial sodium levels below 120 mmol/L [1, 6, 10], a few reports have documented ODS occurring with milder degrees of chronic hyponatremia [7, 8]. Oke et al. described a case of ODS in a 49‐year‐old woman with chronic alcoholic liver disease, a baseline sodium level of 126 mmol/L, and hypokalemia (potassium 3.0 mmol/L) [7]. Additionally, a database analysis of patients undergoing orthotopic liver transplantation identified 10 cases of ODS occurring post‐transplant, with a mean baseline sodium level of approximately 129 mmol/L [8]. Interestingly, ODS has also been reported in a patient with hypokalemia but without hyponatremia, who had multiple risk factors for ODS, including malnutrition and abrupt osmotic shifts caused by initial diuresis followed by aggressive hydration [9]. These observations suggest that avoiding rapid changes in plasma osmolality is crucial for preventing ODS, rather than focusing solely on the correction of isolated hyponatremia as the primary factor contributing to its development.

In this case, the patient received 1.5 L of isotonic saline over several hours and was subsequently discharged without reassessment of serum sodium levels. While the administration of this volume of fluid might appear benign in a patient presenting with vomiting and dehydration, the consequences of sodium correction were unexpectedly severe. Managing fluid therapy for hyponatremia presents significant challenges, as inadvertent overcorrection is relatively common. The formulas designed to estimate serum sodium changes in response to specific fluid regimens often fail to reliably predict outcomes, largely due to the unpredictable effects of water diuresis [11]. In the present case, it is plausible that restoration of the patient's volume status through intravenous hydration suppressed antidiuretic hormone (ADH) release, resulting in increased free water excretion and a subsequent rise in serum sodium levels. However, this hypothesis cannot be definitively confirmed, as serum sodium levels were not re‐evaluated following IV hydration, and urine sodium and osmolality were not measured before or after treatment. Moreover, risk factors in this patient including chronic alcoholism, low phosphorus, and mild hypokalemia may have impaired cerebral osmolyte adaptation, making the brain more susceptible to even mild osmotic shifts. Interestingly, ODS has also been reported in patients with normonatremia who had other significant comorbidities such as chronic kidney disease, liver failure, and chronic alcoholism [12, 13]. Consequently, physicians should be cognizant of this uncommon disease and exercise caution when adjusting sodium levels to mitigate its occurrence. When correcting electrolyte imbalances, it is crucial to be cognizant of the type of hyperosmolar agent and the degree of hyperosmolarity.

ODS is a multifaceted condition with a broad clinical spectrum and significant diagnostic overlap with other neurologic or metabolic disorders. In this case, the patient presented with tremors and ataxia initially attributed to alcohol withdrawal. However, the persistence and progression of symptoms, including truncal ataxia, lower limb weakness, and dysarthria, beyond the expected resolution window of alcohol withdrawal of 5–7 days, and despite appropriate benzodiazepine therapy, argued against this etiology. Hypophosphatemia was also present, but it was not severe and did not correlate temporally with the symptom trajectory or improve with repletion. The subacute onset of symptoms, in conjunction with the characteristic MRI findings involving the central pons and the exclusion of vascular, structural, and metabolic causes, strongly supported ODS as the primary diagnosis [5, 14]. Similar diagnostic challenges have been reported in patients with chronic alcoholism and coexisting electrolyte disturbances, reinforcing the importance of clinical vigilance in high‐risk populations [13, 15].

This case was further complicated by the incidental finding of segmental pulmonary embolism and subsequent anticoagulation with rivaroxaban. While pulmonary embolism itself does not contribute to ODS pathogenesis, its presence influenced the diagnostic approach, leading to misattribution of neurological symptoms to embolic infarction. Additionally, neurological deficits may have predisposed the patient to an unrecognized fall, which, along with anticoagulation, likely contributed to the development and progression of the subdural hematoma. Ultimately, the hematoma expanded, causing midline shift and necessitating neurosurgical decompression. These complications played a pivotal role in the patient's decline and cardiac arrest.

Radiologically, both ODS and ischemic stroke may demonstrate diffusion restriction on diffusion‐weighted imaging (DWI) due to cytotoxic edema. However, acute pontine stroke often exhibits imaging patterns reflective of the vascular territory involved, whereas ODS typically manifests as changes confined to the central pons, sparing the periphery. Notably, these radiological changes in ODS may not become apparent until 2–4 weeks after sodium correction. In this case, the initial MRI revealed a small pontine lesion suggestive of acute infarction; however, image interpretation was compromised by substantial motion artifacts. These artifacts obstructed visualization of lesion margins and distribution, impeding accurate differentiation between stroke and osmotic demyelination. Given the patient's concurrent pulmonary embolism and embolic risk, an ischemic etiology was initially favored. This led to a delay in suspecting ODS and deferral of repeat neuroimaging. On follow‐up MRI, acquired with enhanced image quality, the lesion exhibited a classic “batwing” appearance—central pontine hyperintensity sparing the ventrolateral fibers and corticospinal tracts. The “batwing” appearance, observed in our case, is characteristic of ODS but not pathognomonic. It reflects the selective vulnerability of central pontine myelin to osmotic stress, while peripheral fibers are relatively preserved [16]. This imaging pattern contrasts with pontine infarction, which typically follows vascular territories and may manifest diffusion restriction, contrast enhancement, or extension to peripheral regions. In contrast, ODS lesions frequently lack enhancement and present with symmetric central involvement. Nevertheless, these features may be absent in early stages, underscoring the significance of repeat imaging when clinical suspicion persists and initial scans are inconclusive.

This case has several limitations. First, although the patient was treated for mild pancreatitis and hyponatremia in the emergency department, serum sodium was not rechecked prior to discharge. The next available value was obtained several days later in the outpatient setting, limiting our ability to assess for possible transient overcorrection during the early post‐treatment phase. Second, the absence of urine sodium and osmolality measurements precluded assessment of renal water handling and the role of potential water diuresis due to ADH suppression. Third, early neurological symptoms were misattributed to alcohol withdrawal and embolic stroke, delaying the diagnosis of ODS. Lastly, while the subdural hematoma was surgically managed, its etiology remains unclear, possibly related to anticoagulation, a fall secondary to neurologic deficits, or spontaneous hemorrhage.

Despite these limitations, this case report presents several pertinent clinical insights. First, it enhances the awareness of the risk of ODS in patients with mild hyponatremia who are discharged from the emergency department. Second, the case highlights the challenges associated with managing fluid therapy for hyponatremia, emphasizing the potential for overcorrection even in patients who may not have received substantial intravenous hydration or hypertonic saline. Third, the management of hyponatremia should encompass risk assessment to identify patients at higher risk for ODS and meticulous monitoring of sodium levels. Lastly, considering the delayed and nonspecific presentation of ODS, attending physicians should remain vigilant for early identification and intervention of this debilitating condition.

Osmotic demyelination syndrome (ODS) is a potentially fatal neurological complication that can occur even with modest hyponatremia correction, particularly in high‐risk individuals such as those with chronic alcoholism, malnutrition, and electrolyte disturbances. While the correction rate may appear within acceptable thresholds, impaired cerebral osmotic adaptation may predispose such patients to demyelination.

In this case, the patient's clinical deterioration was multifactorial. An initial misdiagnosis delayed appropriate supportive care and monitoring. Progressive neurological decline, compounded by anticoagulation‐related subdural hematoma, likely related to impaired mobility and increased fall risk, ultimately led to brain herniation and death. This sequence underscores the need to recognize the complex and cumulative risks that can amplify adverse outcomes in ODS.

To reduce the likelihood of such outcomes, hyponatremia management should involve individualized risk assessment, cautious fluid administration, and close monitoring of serum sodium and electrolytes, both during and after correction. In patients with persistent or evolving neurological symptoms, clinicians should maintain a high index of suspicion for ODS and pursue timely neuroimaging to avoid delays in diagnosis and intervention.

## Author Contributions

**Khaled Alsa'ed:** conceptualization, writing – original draft. **Soud Deek:** data curation, writing – original draft. **Gi Eun Kim:** methodology, writing – original draft. **Muhammad Faisal Wadiwala:** data curation, visualization. **Anwar I. Joudeh:** writing – review and editing.

## Ethics Statement

Ethics committee approval was waived.

## Consent

Written informed consent was obtained from the patient for publication.

## Conflicts of Interest

The authors declare no conflicts of interest.

## Data Availability

The data sets used and/or analyzed during the current study are available from the corresponding author on reasonable request.
